# Relation of Biospeckle Activity with Quality Attributes of Apples

**DOI:** 10.3390/s110606317

**Published:** 2011-06-14

**Authors:** Artur Zdunek, Justyna Cybulska

**Affiliations:** Institute of Agrophysics, Polish Academy of Sciences, Doswiadczalna 4, Lublin 20-290, Poland; E-Mail: j.cybulska@ipan.lublin.pl

**Keywords:** biospeckle, firmness, soluble solids content, titratable acidity, apple, starch, nondestructive

## Abstract

Biospeckle is nondestructive optical technique based on the analysis of variations of laser light scattered from biological samples. Biospeckle activity reflects the state of the investigated object. In this study the relation of biospeckle activity (BA) with firmness, soluble solids content (SSC), titratable acidity (TA) and starch content (SC) during the shelf life of seven apple cultivars was studied. The results showed that the quality attributes change significantly during storage. Significant and pronounced positive correlation between BA and SC was found. This result shows that degradation of starch granules, which could be stimulated to vibration by intracellular cyclosis, causes a lesser number of laser light scattering centers and results in smaller apparent biospeckle activity.

## 1. Introduction

Postharvest softening of apple [*Malus domestica* (Borkh.)] fruit is a serious problem for growers [1]. Hence, there is need to evaluate apple quality at different stages of pre- and post-harvest technology in order to provide product of the best quality to consumers [2]. Recently, a few interesting optical techniques and devices have been developed and successfully used for nondestructive evaluation of fruit and vegetables: Vis/NIR spectrophotometry [3], time-resolved reflectance spectroscopy [4], hyperspectral backscattering imaging [5,6], laser-induced light backscattering [7,8] or chlorophyll fluorescence [9–11]. Nikolai *et al.* [12] have reviewed most of the above techniques, collectively naming them NIR spectroscopy, and have shown their feasibility and areas where more research is still needed.

Biospeckle is another optical technique, but less known, that was introduced for nondestructive evaluation of biological materials about fifteen years ago [13,14]. In the method, coherent laser light illuminates an object of interest. The backscattered light interferes and a speckle pattern is created in an observation plane. If the sample does not show activity, the speckle pattern is stable in time. However in the case of biological samples, the speckle pattern consists of two components: the static one from stationary elements of the tissue and the variable one from moving particles of the tissue. The variable in time speckle pattern is characteristic for biological tissue and has been called as the biospeckle (see AVI supplementary material which presents biospeckle of apple) [13,14]. If material is transparent, as in the case of biological tissues, biospeckle activity provides more complex information from the bulk of an object. Bragga *et al.* [15] have summarized that processes related with movement of the scattering centers in the tissue, such as cytoplasmic streaming, organelle movement, cell growth and division during fruits maturation and biochemical reactions, that are responsible for a certain biospeckle activity. Brownian motions should be considered as a source of biospeckle activity too [14]. The knowledge about biospeckle in relation to fruit and vegetables is still limited. Especially, there is lack of robust calibration for certain pre- and postharvest problems. In general, it has been shown that biospeckle activity changes with an age or with some surface properties, for example an infection of a biological object. On the other hand there is lack of consistent biological interpretation of the phenomena.

So far, attempts to apply biospeckle methods in biological studies include measurements of blood flow in blood vessels [16], viability of seeds [17,18], activity of parasites in living tissues [19,20], analysis of maturation and bruising of fruits and vegetables [13,21,22]. These studies showed that decaying of a tissue conditions caused by age, illness/infection or damage, relates with lower biospeckle activity. Monitoring of apples during shelf life showed that the decrease in firmness correlates with decrease of biospeckle activity [23,24]. Surprisingly, up to now no complex comparison of biospeckle activity with other basic quality attribute for apples has been done. Hence the goal of this study was to check whether biospeckle activity correlates with the basic quality attributes: firmness, soluble solids content, titratable acidity and starch content of stored apple under shelf life conditions.

## 2. Material and Methods

### 2.1. Apples and Storage

Apple [*Malus domestica* (Borkh.)] cultivars: ‘Elstar’, ‘Free Redstar’, ‘Gala’, ‘Gold Milenium’, ‘Melfree’, ‘Rajka’ and ‘Szampion’, purchased from the orchard of the Research Institute of Pomology and Floriculture in Skierniewice, Poland, were used for the experiments. Fruits were harvested at the optimum window for the each cultivar and then stored under a normal atmosphere at a temperature of 2 °C for approximately two months prior to the experiments which were performed on all cultivars simultaneously. For each cultivar, about 36 apples of uniform size and free from visible blemishes were selected for the experiments. Then, apples were conditioned at room temperature for one day before a shelf life program consisting of 1, 3, and 6 days of storage. At each of the three days of the experiments, one-third of the apples was tested. First biospeckle activity was evaluated and then firmness was measured on the same spot on the apple equator. Then apples of a given cultivar were mixed together and the pulp was used for the other destructive tests: starch content (SC), soluble solids content (SSC) and titratable acidity (TA). These tests were performed at days 1 and 6 of the experiments.

### 2.2. Biospeckle

**Figure 1.**
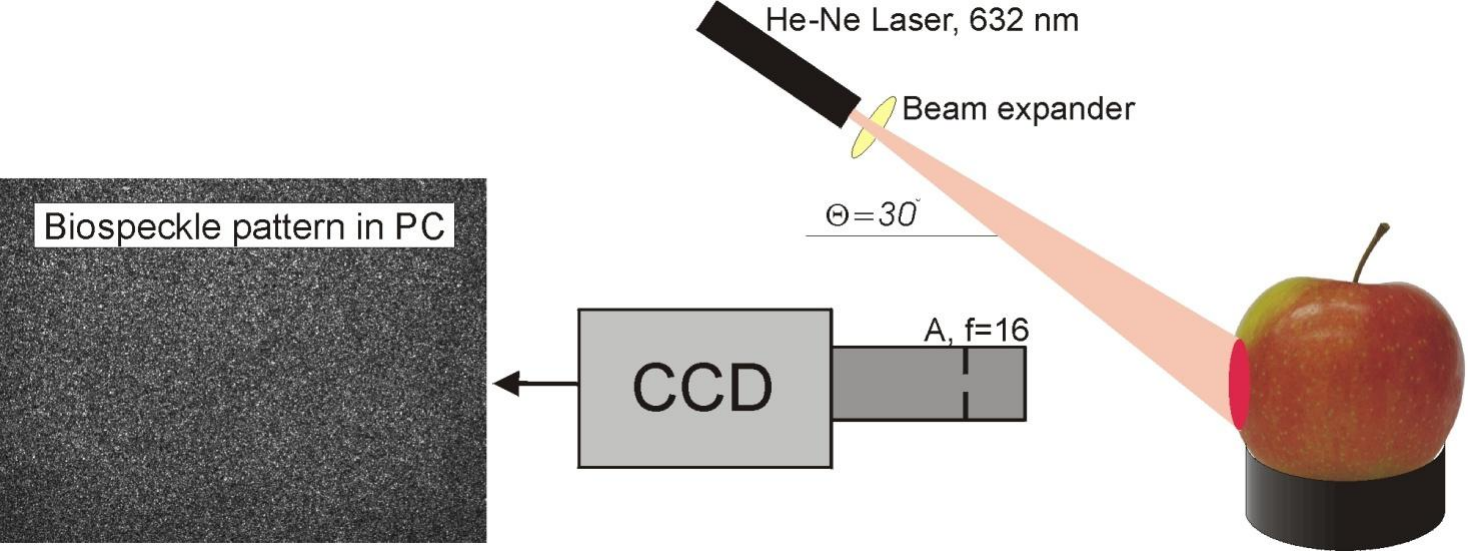
Scheme of experimental setup for biospeckle activity measurement. A—aperture, f—value of objective aperture, CCD—charge-coupled device, θ—angle between laser and CCD. Biospeckle pattern is changing in time for biological objects (see AVI supplementary material).

The biospeckle measurement device was similar to that which was previously used by Zdunek *et al.* [23,24] (Figure 1). The system consisted of a low power He-Ne laser (RBM R. Braumann GMBH, 632 nm, 0.98 mW), with a microscope objective 10X/0,24 (PZO, Poland) as the beam expander to illuminate the sample. Biospeckle were recorded by a CCD camera (Monochrome FireWire Astronomy Camera DMK 21AF04.AS, The Imaging Source Europe GmbH, Bremen, Germany) with a 25 mm defocused objective and a 20 mm extension ring. The camera-object distance was 37 mm and the laser-object one 180 mm. The incident angle was θ ≈ 30 degrees. A stack of uncompressed images (BMP, 8 bits) was recorded during 14 s at a rate of 15 fps. The image size was 640 × 480 pixels, which corresponded to a 32 mm^2^ observation area. Image exposure time of the CCD camera was 1/250 s. Gain and brightness of the CCD camera were optimized experimentally, in order to avoid overexposed pixels on an image histogram. The image acquisition settings were kept unchanged during the whole experiment.

Biospeckle activity was evaluated using the correlation coefficient C^kτ^, where k = 0, 1, 2 … and τ = 1/15 s. C^kτ^ was calculated as the correlation coefficient of data matrix of the first frame (k = 0) with the data matrixes of the following frames (at kτ) from the bitmaps of biospeckle. In this study, C^14^ was analyzed only as the correlation coefficient between the first frame kτ = 0 and the frame at kτ = 14 s. Then, biospeckle activity BA = 1-C^14^ value was determined. Correlation coefficient C^kτ^ was calculated using the Matlab^®^ R2010a software.

### 2.3. Firmness

Firmness (N) was measured with a contact acoustic emission detector (CAED, Institute of Agrophysics PAS, Lublin, Poland) [25,26]. The device has a force sensor of 200 N capacity with accuracy of 0.1% full scale. CAED punctures the fruit with a 11.1 mm diameter probe with a dome-shaped tip with a radius of curvature of 8.73 mm which is inserted 8 mm into the apple at a speed of approximately 20 mm/min. The experiment was performed on peeled apples.

### 2.4. Starch Content

Starch content (SC, %) of apple pulps was determined by the Ewers polarimetric method (ISO 10520:1997) adjusted to low-starch materials. Apple pulp (about 30 g) was weighed in a volumetric flask, suspended in 1.124% HCl (50 mL) and boiled for 15 min. After boiling deionised water (10 mL) was added to the starch suspension which was then cooled. Solutions of 15% K_4_Fe(CN)_6_ (2 mL) and 30% (CH_3_COO)_2_Zn (2 mL) were added to cold suspension, vigorously mixed and topped up with water to 100 mL. The mixture was then filtered, placed in a 200 mm polarimetric tube and the angle of rotation of polarized light was measured.

### 2.5. Soluble Solids Content

Soluble solids content (SSC, Brix) was determined using a digital refractometer (PAL-BX/RI, Atago Co. Ltd., Tokyo, Japan). Apple pulp filtrate was poured onto a prism of the refractometer and soluble solid content was immediately measured. The measurement was performed five times for each sample.

### 2.6. Titratable Acidity

Titratable acidity (TA, g/100g) was determined according to Polish Standard PN-90/A-75101/04. Apple pulp (approximately 40 g) was weighed with 0.001 g accuracy. Deionised water (100 g) was added to the volumetric flask with the pulp. The suspension was heated to boiling and then cooled. Deionised water was added up to 250 mL. After pulp (15 mL) suspension was filtered and water extract (50 mL) were titrated to pH 8.1 with 0.1 M NaOH. The results were calculated as malic acid and expressed as g/100 g apple fresh weight.

### 2.7. Statistical Analysis

Statistical analysis was performed using Unscrambler X 10.0.1 (Camo Process SA, Trondheim, Norway) and Statistica 9.0 (StatSoft, Inc., Tulsa, OK, USA). The mean values and standard deviation (SD) were determined from 12 replicates at each day in the case of biospeckle activity and firmness measurements (for each individual apple). In the case of SC, SSC and TA, three replicates were measured from pulp of 12 apples at day 1 and 6. Principal component analysis PCA on normalized data to their standard deviations was performed and matrix of Pearson’s correlation coefficients (R) was calculated to evaluate the relations between variables. A shelf life effect for individual cultivars was investigated with one-way ANOVA followed by the *post hoc* Tukey’s Honestly Significant Difference (HSD) test. Additionally, shelf life, cultivar and shelf life*cultivar effects were investigated with a two-way ANOVA.

## 3. Results

**Figure 2.**
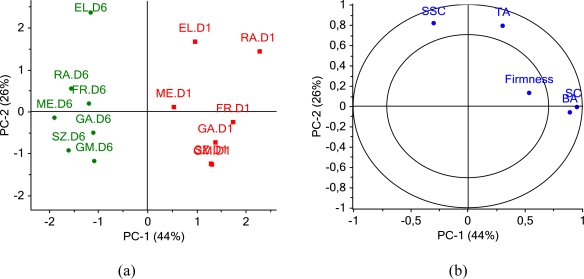
**(a)** PCA scores grouped according to shelf life storage days 1 and 6 for apples: EL—‘Elstar’, FR—‘Free Redstar’, GA—‘Gala’, GM—‘Gold Milenium’, ME—‘Melfree’, RA—‘Rajka’ and SZ—‘Szampion’. D (number)—day of shelf life. **(b)** Variable loadings on PC1 and PC2 axis.

Figure 2(a) presents the PCA plot of scores grouped according to shelf life days. PC1 and PC2 together explain 70% of the samples’ variance. Samples show clear separation according to shelf life storage from right to left on PC1.

Table 1 presents the mean values of apples maturity indexes. Firmness, titratable acidity TA and starch content SC decreased, whereas soluble solids content SSC increased during shelf life. The changes are significant (*p* < 0.05) with the exception of two cases: SSC for ‘Melfree’ and ‘Rajka’ (*p* > 0.05). These results are typical for apples during maturation and postharvest ripening and confirm many revious observations [1,27–29]. These quality attributes are used extensively for prediction of the harvest window or to monitor quality during storage however the main disadvantage is their destructive character. Table 1 shows that biospeckle activity BA decreases during storage similarly to previous results [23,24]. The change of BA is gradual and significant after 6 days of shelf life, with exception of cv. ‘Melfree’ which did not change. After 3 days of shelf life significant change was observed only in a few cases. Similarly, firmness significantly decreased after 6 days in most cases and rarely after 3 days of storage. It implies that BA could be used for nondestructive evaluation of apples quality with accuracy comparable to other techniques.

Figure 2(b) presents loadings of each variable projected on PC1 and PC2. Each variable explains more than 50% of the displayed components. Loading of biospeckle activity BA has the same sign and lies close to starch content SC loading along PC1. It means that sample grouping in Figure 2(a) along PC1 relates with SC and BA. The rest of variables, particularly SSC and TA do not correlate with SC and BA and they both lie along PC2. Table 2 presents the matrix of Pearsons’ correlation coefficients calculated for the whole data set presented in Table 1.

Correlation between BA and SC is significant (R = 0.849, *p* < 0.01), whereas the rest of the mutual correlations are very weak and not significant (*p* > 0.05). It must be emphasized that in this study firmness neither correlated significantly with quality attributes of apples nor with biospeckle activity, despite the significant single-cultivar shelf life effect for the each variable and the same trend with other variables (Table 1). This lack of correlation could be the result of mixing of different cultivars for correlation matrix calculation which was shown by ANOVA as the cultivar effect (Table 1).

On one hand, firmness is a mechanical parameter and may not obviously correlate directly with sweetness or acidity of fruits. On the other hand, even if firmness during storage follows change of SSC, TA or SC, the dynamic may be different for cultivars. Thus in this case, common model for many cultivars for SSC, TA or SC prediction with firmness rather does not make sense.

**Figure 3.**
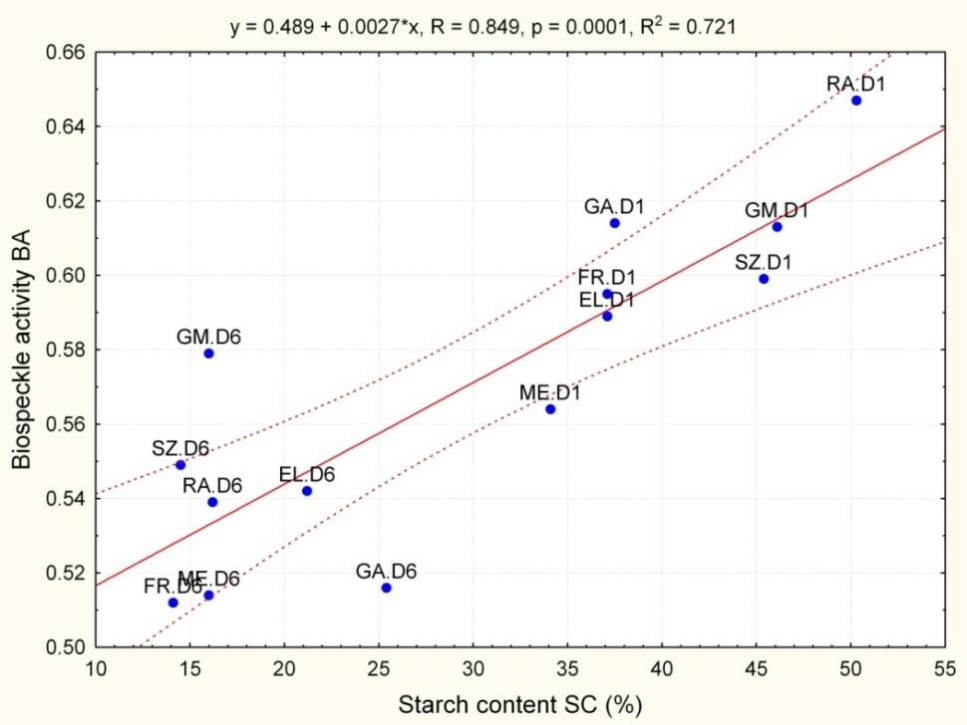
Relation of biospeckle activity BA with starch content SC for apples: EL—‘Elstar’, FR—‘Free Redstar’, GA—‘Gala’, GM—‘Gold Milenium’, ME—‘Melfree’, RA—‘Rajka’ and SZ—‘Szampion’. D (number)—day of shelf life.

From this point of view the significant correlation between BA and SC for the mixed data set for seven cultivars is very interesting. Figure 3 shows that the relation BA *vs.* SC is linear; higher starch content relates with higher biospeckle activity. The points for particular cultivars are randomly located along the linear regression line. The linear trend is clearly visible for apples just removed from cold storage (D1 in Figure 3). It means that biospeckle activity is indeed related with apparent starch content and individual cultivar related properties do not influence on the relationship in this case.

## 4. Discussion

Chloroplasts, other organelles and intracellular particles move together with cytoskeleton in plant cells. The cytoplasmic movement (cyclosis) is needed to maintain optimum conditions for tissue life. Cyclosis causes transports nutrients, enzymes, and larger particles within cells, enhances the exchange of materials between organelles, as well as between cells. In some plant cells the movement is rotary and limited to the peripheral parts of the cell next to the cell wall. This movement may be increased by light, and is dependent on temperature and pH, for example. Laser light is elastically scattered (according to Rayleigh when the target diameter is much smaller than the light wavelength or Mie when the target is similar or larger than the light wavelength) on each boundary, like intracellular membranes, organelles and other particles. If any object due to cyclosis is moving, the scattering causes an unstable biospeckle pattern. From this point of view, the biospeckle activity is a function of particles’ activity (mobility) and vitality of a tissue. Apples, like many other fruit crops, accumulate starch at early stages of maturation and progressively degrade starch to increase sweetness during ripening [30–32]. Starch granules are formed in amyloplasts within cells and have a size from 1 μm to 100 μm. For apples the average size of about 2 to 12 μm was found [31,32]. Large particles within cells obviously affect optical properties of the tissue. Laser light of 632 nm is scattered on starch granules which are bigger than 2 μm according to Mie’s theory. Starch does not move around together with organelles however the cyclosis presumably cause some vibrations of the granules. Thus, apart other moving organelles, starch granules would give many additional non-stationary scattering centers. In result, more starch particles means higher apparent biospeckle activity (Figure 3). Probably some other ripening-related processes like cell wall polysaccharides depolymerisation or malic acid enzymatic degradation would alter laser light scattering inside apple tissue if any particles are actuated in cells, according to hypothesis that even Brownian motion could be the source of non-stationary laser light scattering. However this should be studied more in the future to give consistent evidence.

In this study, chlorophyll content was not measured. There is evidence that less chlorophyll content causes higher apparent biospeckle activity [33] due to light absorption by this pigment and in consequence shallower light penetration through a tissue. In apples, postharvest storage is associated with chlorophyll degradation, hence it gives an opposite effect to the starch effect on BA, *i.e.*, presumably the effects compensate each other. It suggests that if apples used in this experiment contained any chlorophyll, a real starch effect on biospeckle activity would be even more pronounced than this shown in Figure 3.

## 5. Conclusions

This study showed that biospeckle activity for apples depends significantly on starch content and the relation is general for various cultivars, which is not true in the case of firmness, soluble solids content and titratable acidity. Postharvest ripening of fruits is a very complex phenomenon and some processes significantly change the physical composition of the tissue. At present one can summarize this study and previous observations that starch degradation decreases the biospeckle activity of apples whereas chlorophyll degradation causes its increase. Another issue, which has not been studied yet, is what happens with molecules after postharvest pectin or starch degradation: do they contribute to biospeckle activity? Postharvest water evaporation while free air storage presumably would decrease particle mobility, but there is no clear evidence yet to prove this. In summary, it seems that biospeckle method is very promising for nondestructive monitoring of ripening processes however more specific research should be done to obtain a robust calibration due to competitive effects of starch and chlorophyll degradations on biospeckle activity.

## Figures and Tables

**Table 1.** Quality attributes and biospeckle activity of apples during shelf life. SD—standard deviation, BA—biospeckle activity, TA—titratable acids, SSC—soluble solids content, SC—starch content.

**Cultivar**	**Shelf life (days)**	**BA ± SD**	**Firmness (N) ± SD**	**TA (g/100 g) ± SD**	**SSC (°Brix) ± SD**	**SC (%) ± SD**
Elstar	1	0.589 ± 0.037^a^	50.7 ± 5.9^a^	0.701 ± 0.002^a^	14.4 ± 0.4^a^	37.1 ± 3.0^a^
	3	0.556 ± 0.049^ab^	42.1 ± 5.3^b^			
	6	0.542 ± 0.034^b^	40.3 ± 5.2^b^	0.650 ± 0.003^b^	15.5 ± 0.1^a^	21.2 ± 1.0^b^
	*F*-value	4.2*	11.6*	221.0*	19.0*	144.0*

Free Redstar	1	0.595 ± 0.052^a^	66.9 ± 6.7^a^	0.576 ± 0.004^a^	12.7 ± 0.1^a^	37.1 ± 1.1^a^
	3	0.557 ± 0.100^ab^	64.7 ± 7.3^a^			
	6	0.512 ± 0.054^b^	57.1 ± 6.3^b^	0.502 ± 0.001^b^	13.6 ± 0.1^b^	14.1 ± 1.2^b^
	*F*-value	3.7*	6.9*	508.0*	364.5*	1,512.4*

Gala	1	0.614 ± 0.068^a^	69.4 ± 7.6^a^	0.276 ± 0.002^a^	13.9 ± 0.3^a^	37.5 ± 1.7^a^
	3	0.554 ± 0.047^b^	70.6 ± 9.8^a^			
	6	0.516 ± 0.047^b^	56.5 ± 7.4^b^	0.258 ± 0.003^b^	14.2 ± 0.1^a^	25.4 ± 0.3^b^
	*F*-value	9.8*	10.7*	908.8*	2.7^ns^	268.8*

Gold Milenium	1	0.613 ± 0.044^a^	36.9 ± 3.2^a^	0.411 ± 0.002^a^	12.8 ± 0.1^a^	46.1 ± 1.1^a^
	3	0.563 ± 0.050^b^	33.3 ± 3.7^a^			
	6	0.579 ± 0.029^b^	29.0 ± 3.8^b^	0.355 ± 0.003^b^	13.3 ± 0.1^b^	16 .0 ± 0.6^b^
	*F*-value	4.2*	14.6*	415.8*	84.5*	2,209.6*

Melfree	1	0.564 ± 0.064^a^	49.6 ± 9.6^a^	0.582 ± 0.002^a^	13.2 ± 0.1^a^	34.1 ± 0.6^a^
	3	0.537 ± 0.054^a^	44.5 ± 12.2^a^			
	6	0.514 ± 0.045^a^	34.7 ± 4.1^b^	0.426 ± 0.001^b^	13.9 ± 0.0^b^	16 .0 ± 0.6^b^
	*F*-value	2.3^ns^	8.0*	486.7*	400.0*	3,325.5*

Rajka	1	0.647 ± 0.096^a^	52.4 ± 4.6^a^	0.616 ± 0.005^a^	14.7 ± 0.1^a^	50.3 ± 0.6^a^
	3	0.577 ± 0.041^ab^	50.7 ± 5.3^a^			
	6	0.539 ± 0.070^b^	42.3 ± 4.5^b^	0.410 ± 0.00^b^	14.8 ± 0.1^a^	16.2 ± 0.9^b^
	*F*-value	6.4*	15.2*	1,287.6*	1.0^ns^	5,332.2*

Szampion	1	0.599 ± 0.060^a^	46.3 ± 6.5^a^	0.388 ± 0.000^a^	12.9 ± 0.2^a^	45.4 ± 1.7^a^
	3	0.557 ± 0.026^ab^	38 .0± 3.5^b^			
	6	0.549 ± 0.046^b^	30.4 ± 3.9^b^	0.355 ± 0.004^b^	13.5 ± 0.1^b^	14.5 ± 0.9^b^
	*F*-value	3.8*	32.3*	304.3*	36.1*	1,379*

Effect		*F*-value of two-way ANOVA (day 3 excluded from analysis)				

Cultivar		3.03*	88.52*	3858*	132.0*	23.09*
Shelf life		57.7*	161.9*	2705*	133.5*	2423*
Shelf life*Cultivar		1.50	1.50	256.9*	6.8*	44.35*

* means shelf life effect significant at *p* < 0.05. The same superscript letter means no significant difference.

**Table 2.** Pearsons’ correlation coefficient matrix between variables measured in this study for seven apple cultivars. BA—biospeckle activity, TA—titratable acids, SSC—soluble solids content, SC—starch content.

	
	**BA**	**Firmness**	**TA**	**SSC**
**BA**	---			
**Firmness**	0.234	---		
**TA**	0.185	0.100	---	
**SSC**	−0.228	−0.024	0.301	---
**SC**	0.849**	0.412	0.242	−0.237

** means correlation is significant at *p* < 0.01.
